# Movement Behaviour and Health Outcomes in Rural Children: A Systematic Review

**DOI:** 10.3390/ijerph20032514

**Published:** 2023-01-31

**Authors:** Douglas Vieira, Elenir Campelo Gomes, Ângelo Solano Negrão, Mabliny Thuany, Thayse Natacha Gomes

**Affiliations:** 1 Post-Graduation Program of Physical Education, Department of Physical Education, Federal University of Sergipe, São Cristóvão 49100-000, Brazil; 2 Post-Graduation Program of Human Movement Sciences, Federal University of Pará, Belém 66075-110, Brazil; 3 Post-Graduation Program of Anthropic Studies in the Amazon, Federal University of Pará, Belém 66075-110, Brazil; 4 Centre of Research, Education, Innovation and Intervention in Sport (CIFI2D), Faculty of Sport, University of Porto, 4200-450 Porto, Portugal; 5 Department of Physical Education and Sports Science, University of Limerick, V94 T9PX Limerick, Ireland; 6 Physical Activity for Health Cluster, Health Research Institute, University of Limerick, V94 T9PX Limerick, Ireland

**Keywords:** physical activity, sedentary behaviour, health

## Abstract

Background: Studies with rural children are limited, and results are divergent regarding the information on movement behaviours. Purpose: to (i) describe the physical activity and sedentary behaviour in children; (ii) synthetize the year and place of publication, methodological quality, and instruments used to measure physical activity and sedentary behaviour; and (iii) to analyse the relationship between physical activity, sedentary behaviour, and health outcomes in these children. Methods: We use the databases PubMed, Web of Science, SPORTDiscus, Scopus, Virtual Health Library, and SciELO, considering papers published until October 2021. A total of 12,196 studies were identified, and after the exclusion of duplicate, title and abstract screening, and the full-text assessment, a total of 68 were included in the study. Results: A cross-sectional design was dominant among the studies, with sample sizes ranging from 23 to 44,631 children of both sexes. One-third of the studies were conducted in North America and Europe, and most of them used device-based measurements. Inequalities were observed regarding sex, age, economic level, race, and physical activity domains within and between the places of residence. Sociodemographic characteristics were also related to health outcomes for children living in rural and urban areas. Conclusion: It is necessary to increase the evidence on movement behaviours among children living in the countries of South America and Oceania, as well as to increase the level of evidence on the role of school for physical activity in children in rural areas, given the inconsistent findings.

## 1. Introduction

Children’s movement behaviour is closely related to environmental aspects [1,2], which include not only the physical environment (e.g., urbanization, housing infrastructure, climate, and transport) [3], but also the social (e.g., family support, encouragement of friends and partners), cultural, and the political ones [2]. Considering the physical environment, previous studies showed differences in physical activity levels between children from urban and rural areas [4,5]. In general, a reduced number of structured spaces for physical activity practices, safety perception, motorized transport, and high availability of screen leisure activities are pointed out as factors related to a decrease in physical activity levels among children living in urban areas [6,7,8]. On the other hand, rural areas may have a more peaceful lifestyle, with more open-air areas, greater possibilities for active transport, and even aspects related to labour activities (generally involving manual work), which can play a relevant influence on children’s movement behaviours, with a positive impact on their physical activity levels [9].

Physical inactivity and a high amount of time in sedentary behaviours are associated with an increased risk of developing non-communicable diseases (e.g., hypertension, diabetes, and cancer) [10], increased body adiposity, with a negative impact on health and physical fitness in children [11,12]. In this context, for health benefits, the World Health Organization (WHO) suggests that children and adolescents (aged 5–17 years) should be engaged in at least an average of 60 min/day in moderate to vigorous physical activity and also limit the time spent on sedentary behaviour, especially recreational screen time [13,14]. 

Based on this, it seems of relevance to better understand the role of the environment on children’s movement behaviour, and how this relationship impacts their health. However, most of the previous studies focusing on investigation of correlates and/or determinants of physical activity and sedentary behaviours in children have been developed by sampling youth from urban areas [15,16,17,18,19]. Studies with rural children are limited [9], and results are divergent regarding the compliance with WHO physical activity guidelines, as well as physical activity and sedentary behaviour predictors, and their association with health outcomes [20]. 

In addition, although the increase in urbanization, recent estimates show that more than 50% of children worldwide live in rural areas, and these children often live in less favoured conditions than their urban peers [21], highlighting the need for more attentive support for this group, especially related to their general health. It should be noted that researches that evaluate children’s health (or development in general) usually come from western countries, also called minority, which sometimes ends up generalizing and creating interventions to other countries or regions (majoritarian) based on their own reality, disregarding much broader and diversified contexts/environments [22].

Therefore, it seems of relevance to summarize the published studies that addressed this topic, to better understand the role of the rural environment on movement behaviours, and its association with child health. So, considering studies that sampled children living in rural areas, this systematic review aimed to (i) synthetize the year and place of publication, methodological quality, and instruments used to measure movement behaviours (physical activity and sedentary behaviour); (ii) describe physical activity and sedentary behaviour in children from rural settings; (iii) analyse the relationship between physical activity, sedentary behaviour, and health outcomes in these children; and (iv) present the differences between rural and urban children regarding movement behaviour and health indicators, when this information was presented in the studies.

## 2. Materials and Methods

### 2.1. Protocol

This systematic review was performed using the Preferred Reporting Items for Systematic Reviews and Meta-Analyses (PRISMA) guidelines [http://www.prisma-statement.org/ (accessed on 17 September 2022)] being previously registered at the International Prospective Register of Systematic Reviews (PROSPERO, CRD42021283549) [https://www.crd.york.ac.uk/prospero/ (accessed on 17 September 2022)].

### 2.2. Databases and Search Strategy

The search process was carried out in the following electronic databases: PubMed, Web of Science, SPORTDiscus, Scopus, Virtual Health Library (BVS—*Biblioteca Virtual em Saúde*), and SciELO, considering papers published until October 2021. Based on the PICO strategy, the search terms and Boolean operators used in the search strategy were: (child* OR school* OR young* OR kids) AND (“physical activity” OR exercise OR sport* OR “physical inactivity” OR sedentar* OR sitting OR “screen time”) AND (health) AND (rural). The search was performed using the terms in English, Portuguese and Spanish, and the truncation symbol (*) was used in some terms with the purpose to provide a wider scope within the context of the search. All the results found in the databases were downloaded and uploaded to the reference manager EndNote software (version X9.0, Clarivate Analytics, Philadelphia, PA, USA), which was used during the screening procedures.

### 2.3. Inclusion and Eligibility Criteria

To be included in the study, papers were required to fill the following criteria: (i) original papers; (ii) published in English, Portuguese or Spanish; (iii) sampling children up to 14 years old, from, even partially, rural areas (studies whose sample age exceeded the age of 14 y were also included, but results were only considered, for this review, from those aged ≤14 y); (iv) with the purpose to assess physical activity and/or sedentary behaviour (with health-related benefits). Further, no restriction regarding study design or publication year was considered.

### 2.4. Study Selection

Searching in the databases and screening processes were performed by two researchers, independently. First, search results from each database, were downloaded and uploaded to Endnote software, and duplicate results were excluded. Further, using the previously established eligibility criteria, papers were firstly selected based on their title and abstract, and the remaining papers were read in full text. At this stage, in case of divergences between the two reviewers, a third researcher was consulted.

### 2.5. Quality Assessment

The studies included in this research were evaluated for their methodological quality by two independent researchers. The checklist used (Table 1) was adapted from a previous published study [23], which comprises 10 items, which evaluate: (1) the study aims; (2) sample characteristics; (3) sample size justification; (4) instruments for data collection; (5) statistical analysis; (6) description of the results; (7) conclusions; (8) practical implications; (9) limitations; and (10) directions for future research.

If divergences in the evaluation were found between the two researchers, a third researcher evaluation was considered. Selected articles were not excluded based on the results of this evaluation. Based on this evaluation, the studies were categorized into levels: high (>75%), intermediate (50–74%), and low (<50%).

## 3. Results

### 3.1. Included Studies

**Figure 1:**
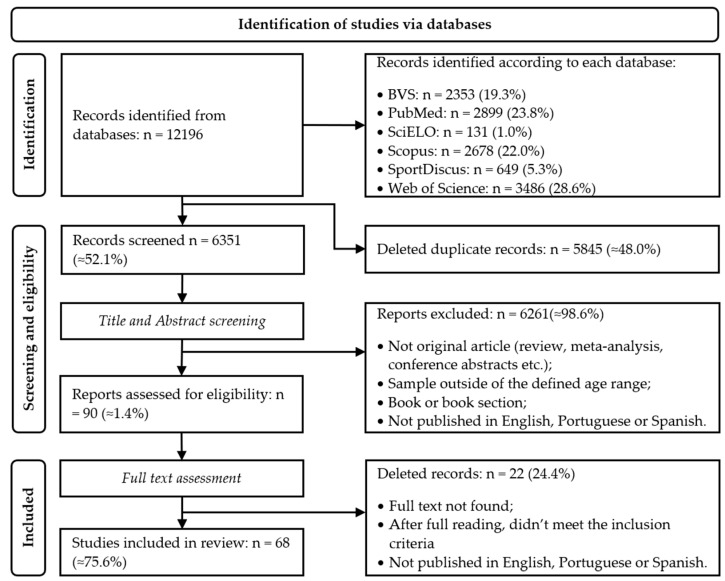
Flowchart for data screening (adapted from PRISMA 2020 flow diagram [24]).

Figure 1 shows the study selection process flowchart. A total of 12,196 studies were identified. Duplicate records were deleted, using EndNote’s specific tool, and then through a manual check, resulting in 6351 studies for title and abstract screening. After reading the title and abstract, ≈98.6% of the papers were excluded, and 90 were fully assessed, from which 68 were included in the study.

Table 2 (rural) and Table 3 (comparison between rural and urban) present data extraction from the selected papers. Information is related to the sample location (continent, country, and specific region, when informed), sample characteristics (size and age/school grade), instruments used (based-device or questionnaires), main results, and the evaluation of methodological quality.

### 3.2. Methodological Quality Assessment

The methodological quality attributed to the studies were categorized based on the study of Abarghoueinejad et al. [23]. No research presented a low methodological evaluation. In general, most were evaluated as high methodological quality (62), and only seven were classified as intermediate. Among the studies that sampled only rural children, four of them were classified as intermediate, while 33 were classified as high (only one study [40] reached the highest score). Among the studies that compared urban vs. urban subjects, two of them obtained a grade that classified them as intermediate (one with 50% [68]), and 29 of them were classified as high (of which reached the highest score [4,62,73,74]).

### 3.3. General Aspects

All the studies—except two [Benefice et al. [25] (longitudinal) and Franco et al. [77] (mixed-longitudinal)]—used a cross-sectional design. The majority of the studies sampled children of both sexes, with one of them sampling only girls [79], aged between two to 19. Further, the sample size ranged from 23 [60] to 44,631 [81] subjects—13.3% had up to 99 subjects, 45.5% sampled between 100–499 individuals, 13.3% sampled between 500–1000, 27.9% sampled more than 1000 participants.

Regarding the year of publication, the oldest study included in this review dates from 1997 [42], and from this year until the end of the first decade of the 21st century, 20 (29.4%) studies were identified [25,26,27,37,42,43,44,45,46,58,63,69,70,71,72,79,80,81,84,85]. However, since then, an increase in the number of publications was observed, and between 2016–2021 we were able to identify 25 (36.7%) published studies that attained our eligibility criteria, i.e., in these last five years there was a higher number of publications than in the first 13 years considered in the reviewer timeframe [4,6,32,33,34,35,36,40,41,52,53,54,55,56,57,61,62,67,68,76,77,78,87,89,90].

Regarding the place where studies were conducted, almost one-third of them [22 (32.3%)] sampled children from North America (USA [4,42,43,44,45,46,47,48,49,50,51,53,55,57,79,80,81,82,83] and Canada [52,54,56]). Europe was the second continent with the highest number of publications, with 15 (22.0%) studies (Portugal [40,74,75,76], Spain, [39,71,77], Cyprus [69,70], Poland [38,41], United Kingdom [73,78], Greece [72], and Ireland [37]); followed by Asia [12 (17.6%) studies—China [35,63,68], India [65,67], Japan [33,36,64], Malaysia [31,34], Iran [66], and Nepal [32]]; and Africa [9 (13.2%) studies—Mozambique [26,27,30], South Africa [6,29], Kenya [28,62], Senegal [25] and Uganda [61]]. South America [Brazil [59,88], Chile [60,89], and Ecuador [90]] and Oceania (Australia [58,84,86,87] and New Zealand [85]), had 5 studies each (14.7% together).

### 3.4. Instruments Used

Among studies that sampled only children from rural settings, both questionnaires and device-based measurements were used to estimate physical activity and/or sedentary behaviour. Of these studies, 14 of them used device-based measurements, namely accelerometers [25,27,29,32,37,40,45,48,52,53] or pedometers [6,28,31,44], which were mostly conducted in North America or Europe, with sample sizes, in general, smaller than studies that used questionnaires (maximum with 406 subjects). In general, movement behaviours were estimated based on 7 days of device use (ranging from 3 to 7 days). 

Regarding the studies that used questionnaires (20), different instruments were used (such as Physical Activity Checklist, Physical Activity Questionnaire for Children, IPAQ-A, Self-Administrated Physical Activity Checklist, Youth Activity Profile, PAQ-C, System for Observing Fitness Instruction Time, Previous Day Physical Activity Recall) [26,30,33,34,35,36,38,39,41,42,43,46,47,49,50,51,57,58,59,60], as well as different strategies to describe the studied variables—for example, specific regression equations, the use of METs (metabolic equivalent), means and medians of counts and predefined cut-off points. In addition, three studies used accelerometers and questionnaires together [54,55,56], providing additional information regarding the type of activities children were enrolled in, as well as food consumption and socioeconomic status.

On the other hand, the majority of the studies that sampled both rural and urban/suburban children used questionnaires to estimate physical activity and/or sedentary behaviour [4,43,61,63,65,66,67,68,70,71,72,76,77,79,80,81,82,84,87,88,89,90]—only 8 of them used device-based measurements, such as an accelerometer [62,64,73,74,75,78,83] or pedometer [69] (most of them conducted in Europe). Only one survey combined the use of questionnaire and pedometer [86]. In addition, a large variation in sample sizes was also observed among these studies, ranging from 129 to 44,631 children/adolescents. 

### 3.5. Physical Activity

#### 3.5.1. Rural Sample

Among studies that sampled only rural children, some aimed to investigate possible disparities in physical activity related to sex and age. In general, boys were more active than girls [6,27,28,29,31,36,38,39,42,43,45,48], and also spent more time in moderate physical activity (MPA) [32] and vigorous physical activity (VPA) [27,32,33,37,40,43,56,57]. However, three studies reported that girls were more active than boys [26,30,60], and in four papers non-differences were found in physical activity according to sex [34,44,49,58].

Regarding age, from the published studies, it was possible to notice that the intensity and/or time (minutes) of physical activity decreases with increasing age [27,43,45,57], but one study found the opposite—lower levels of physical activity among young children compared to their oldest peers [42].

About the domains of physical activity, two studies reported the role of domestic tasks in the amount of physical activity of youth from rural areas, especially among girls [26,27]. On the other hand, regarding the leisure domain, boys were more active than girls, due to more involvement in sports practice [26]. Moreover, results related to active transportation showed that children who walk to school presented a higher daily steps average than those who reported using some type of inactive transportation [28], and scholars who lived close to schools used more active commuting to go to/from school [39]. 

A trend related to differences in physical activity was not observed when comparing school days and weekend days. For example, among children, Fukushima et al. [36] reported that children were more active on days without classes, while Button et al. [56] found the opposite—children were less active on weekends, and Brazendale et al. [55] did not find differences in physical activity among children based on days of the week. Among adolescents, Williams et al. [32] reported that teenagers were more active during school days.

Only one study investigated the role of the weather in youth physical activity, showing a positive association between increasing temperature and an increase in time spent in MVPA [56] among Canadian children. Regarding the role of the time in children’s physical activity, the longitudinal study conducted by Benefice et al. [25] analysed three cohorts of Senegalese children between 13 and 15 years old (1997, 13 years; 1998, 14 years; and 1999, 15 years). The results from the last cohort (1999) showed (compared to the other first two cohorts) a decrease in MPA levels and an increase in VPA, but no significant changes were observed for LPA [25].

#### 3.5.2. Rural × Urban Samples—Comparison

Studies that compared children from rural × urban/suburban areas showed, in general, that rural children were more active than their urban/suburban peers [62,65,66,72,77,78,80,81,85,86,87], with only one study showing the opposite—children from a rural area had a lower steps count compared to those from an urban area [64]. This pattern was also observed when stratified by sex, where rural girls were more active [74,81] and spent more time in MVPA than the urban ones [75,83,86]. Only one survey showed rural boys as being more active than urbans [75]. 

However, some studies did not observe differences in physical activity (time and/or level) according to sex [73] and place of residence [4,70,71,79]. In addition, regardless of the place of residence, some studies showed that boys were more active [80,89], and only one study found high activity levels among girls when compared to boys [77]. Regarding race, one study showed that, among rural children, black girls were more vigorously active, and when comparisons between rural vs. urban were made, white girls were less engaged in VPA [79].

Regarding active transportation, Andrade Neto et al. [88] and Itoi et al. [64] described that rural children tend to use less active commuting to/from school than urban children, while Kundapur et al. [67] reported, specifically about the use of bicycle to go to school, that rural children used more this commuting strategy compared to urban ones, and Christiana et al. [4] and Morais Macieira et al. [76] found that, considering active commuting as a whole, rural youth tend to be more active to go to/from places. Moreover, domestic activities, such as fetching water and herding animals, were more usual among rural samples, playing relevant roles in their total physical activity [61]. About sports practice, Bathrellou et al. [70] observed a lower involvement of rural children in sports, compared to urban ones.

Considering the period of the day, Kundapur et al. [67] and Joens-Matre et al. [80] found that rural children and adolescents engage more in physical activities at night than their urban peers. Moreover, the role of seasons was investigated in two studies, with divergent results: Loucaides et al. [69] showed that rural children take more steps in summer and fewer steps during winter, and spent more time outdoors in general than their urban peers [69]; while McCrorie et al. [78] reported that rural youth spent more time in MVPA in winter and lower levels in spring than urban youth.

### 3.6. Sedentary Behaviour

#### 3.6.1. Rural Sample

Studies with rural children observed some sex differences—boys spent less time in sedentary behaviour than girls [37,45,49,54]; but one study pointed out that boys spent more time on computer games [38]. Regarding age, two studies reported that older children and adolescents spent more time in sedentary behaviour [53,54,59].

Regarding sedentary behaviour on school days, Brazendale et al. [55] observed that children were less sedentary during school days and had more screen time during the week, while Pate et al. [42] stated that students who watched television or played video games for more than 3 h after school were more likely to be inactive [42].

Furthermore, two studies reported results regarding race and socioeconomic aspects. Newton et al. [48] showed that children with low socioeconomic status spent less time in sedentary behaviour, and Moore et al. [46] reported that black children spent more time watching TV during school days when compared to white children [46].

#### 3.6.2. Rural × Urban Sample—Comparison

Studies that compared children living in rural and urban settings observed, in general, that rural children spent less time watching TV and/or using computer [4,62,65,66,68,78,84,85,90], notwithstanding Andrade Neto et al. [88] have pointed out that rural children spend more time watching TV, and Morais Macieira et al. [76] reported that among children who spent >2 h/day on screen, the majority of them were from a rural area. One study did not find differences in screen time between rural and urban youth [70].

When the compliance of screen time guidelines was investigated, rural children complied the most when compared to urbans [62,87]. Taking into account sex differences, results seem to be quite different, although Dollman et al. [86] have noticed that rural boys spent less daily screen time than urban ones. In addition, the two studies from Machado-Rodrigues et al. [74,75] revealed different results, due to the use of different instruments to collect information about the same variable. For example, using accelerometers, authors found that urban girls spent more time in sedentary behaviour than rural ones [74], while through the use of questionnaire, and focusing on screen-time, authors reported that rural girls had more screen-time sedentary behaviour [75]. Dollman et al. [86] did not find any difference in TV time between urban and rural girls [86]. 

### 3.7. Health Outcomes

#### 3.7.1. Rural Sample

Overall, the prevalence of overweight and obesity in children/adolescents ranged from 16% to 38% [6,31,38,39,40,41,47,49,50,51,55,59,60], but in one study this frequency was lower than 8% [35] while in another one the frequency was close to 50% [53]. Regarding sex-differences, different results were founded, with some studies highlighting a higher frequency of overweight/obesity among girls [6,40,50], one study showing a higher frequency among boys [38], and no differences were observed in three studies [44,49,53].

Regarding the association between movement behaviours and health outcomes, several studies showed significant associations between physical activity and sedentary behaviour with BMI [6,25,41,53] (more time spent in sedentary behaviour was related to a higher BMI). In addition, the involvement in physical activity seems to differ accordingly to children’s fat percentage or nutritional status, since girls with high fat percentage were more physically inactive than their peers with lower levels of body fat [45]. Shriver et al. [49] and Bin Saad et al. [34] showed that between 70% and 80% of overweight/obese children were inactive [34] or spent less time in moderate physical activity compared to normal-weight children [49].

Some studies investigated other health outcomes in addition to BMI. Moore et al. [46] reported that children with low levels of physical activity were three times more likely to have metabolic syndrome and twice more likely to be overweight than children with high physical activity levels. On the other hand, Santos et al. [30] pointed out that no significant associations were observed between levels of physical activity and cardiorespiratory fitness.

#### 3.7.2. Rural × Urban Comparison

Different results were shown for children from both rural and urban areas. Almost all the studies analysed overweight as a health outcome, and results were different, showing (i) non-differences in nutritional status according to the place of residence [70,71,76], or (ii) higher prevalence of overweight/obesity among rural children [74,80,81,82], or (iii) higher prevalence of overweight/obesity [63,89,90] and higher levels of subcutaneous adipose tissue [85] among urban children.

Regarding differences taking into account sex and the place of residence, one study reported a higher prevalence of overweight among rural boys [81], while another showed a higher prevalence of obesity among urban girls [89], and one study stated no differences between urban and rural girls for overweight prevalence [81]. Some studies investigated health outcomes according to physical activity and sedentary behaviour. Liu et al. [81] observed that among physically active children, those living in rural areas had higher levels of overweight. In addition, the study conducted by Xu et al. [63] showed that children who spent more time in sedentary behaviour (tv time >7 h/week), regardless of where they live, were more likely to be overweight.

## 4. Discussion

**Figure 2:**
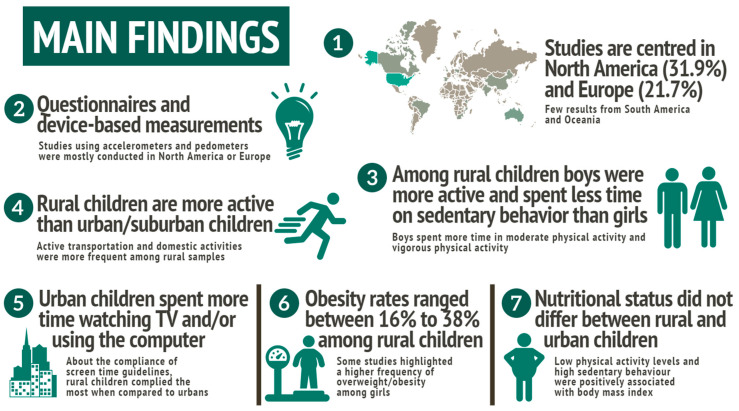
Summary of the main findings.

The purposes of this systematic review were (i) to describe physical activity and sedentary behaviour in children from rural settings; (ii) synthesize the year and place of publication, methodological quality, and instruments used to measure movement behaviours (physical activity and sedentary behaviour); and (iii) to analyse the relationship between physical activity, sedentary behaviour and health outcomes in children living in rural areas. Substantial and methodological findings are highlighted: (i) little evidence is available from longitudinal studies, (ii) studies are centred in North America and Europe, with few results from South America and Oceania; (iii) both device-measurement and questionnaire were used for data collection; (iv) inequalities regarding sex, age, economic level, race, and physical activity domains within and between the places of residence (i.e., rural and urban areas); (v) sociodemographic characteristics were also related to health outcomes for children living in rural and urban areas. Figure 2 highlights the seven main findings of this systematic review.

### 4.1. Physical Activity and Health-Related Outcomes in Rural Children

Inequalities in physical activity were found among children living in rural areas. The role of sex and age was established, since older children tend to present less time in physical activity. These results are in accordance with previous studies [91,92,93]. Inconsistent findings were found regarding sex differences, except for physical activity in the domestic activities domain, in which girls presented higher mean values comparatively to boys [26,27], and physical activity during leisure time, in which boys were more active [26]. Differences in physical activity between sexes are similar to previous findings [94]. In summary, these differences were related to social expectations and cultural stereotypes, that overvalued the role of girls in domestic activities [26]. The paradox of physical activity was previously tested among adults [95] and the results showed that resources and environmental factors are the main barriers to PA practice, while social influences are the main motivator for involvement and adherence to physical activity; however, future studies need to deeply understand the characteristics of the domestic activities performed by children living in rural areas, especially the health-related outcomes possibly associated with the involvement of these activities. 

Obesity rates ranged from 16% to 38% among children living in rural areas, and similar results were observed among urban children (19% to 36%) [6,31,38,39,40,41,47,49,50,51,55,59,60], with inconsistent findings for sex [6,38,40,44,49,50,53]. Low physical activity levels and high sedentary behaviour were positively associated with BMI [6,25,41,53]. The direction of this relationship is well established in the literature [96]. The negative spiral of disengagement [97] refers to the risk of obesity when the relationship between physical activity and motor competence was not well developed during the first years of the children’s life. These findings were supported by recent studies [10,11,12], and different health outcomes were investigated [96,98]. For the present review, among the revised studies, metabolic syndrome was also investigated among children living in rural areas [46], with no association be observed with physical fitness [30]. The association between BMI and physical activity needs to be investigated in longitudinal design studies sampling rural children, due to the relevance of the outcome derived from this relationship to people’s health across the lifespan, and also since this relationship may change from childhood to adolescence. In addition, the mentioned relationship, and its outcomes, can be presented differently in children living in rural settings compared to results derived from children living in urban areas.

Moreover, differences in physical activity according to days of the week showed different results, with some studies showing that children were more active on days without classes [36], while Button et al. [56] found the opposite, i.e., children were more active during school days. These differences may be related to the environmental characteristics considered, as well as different methods used in the studies [36,56]. Therefore, living near the school was positively associated with active commuting [39]. Previous results showed that long distances to be covered during commuting and household income were negatively related to active transportation among children [99], while recreational facilities and the existence of walking or bike paths increase the use of active transportation [99]. Notwithstanding the relevance of active commuting and leisure physical activity for children’s health, specific environmental conditions must be taken into account, since the natural and built environments can affect, both positively or negatively, the involvement in physical activity in its different domains. For example, as shown, in the Canadian context it was observed that higher temperatures were related to higher physical activity levels among children [56] (each 1 °C (1.8 °F) increase in temperature leads to increases in MVPA by about 1.2 min) due to opportunities to perform physical activities in outdoor spaces. However, this relationship must be better explored in countries with higher temperatures during most of the year, which could allow the understanding of the role of weather in active commuting and outdoor leisure physical activity. 

### 4.2. Differences in Physical Activity and Health-Related Outcomes between Rural and Urban Children

The main findings showed that children from rural areas presented, in general, higher physical activity levels [62,65,66,72,77,78,80,81,85,86,87] as well as were more active in the domestic domain [61], regardless the sex. Differences in physical activity, such as during leisure, are related to environmental features, such as security perception and the availability of outdoor spaces for physical activity [100], which seems to be friendly/available in rural areas. In addition, differences in physical activity in the domestic domain can be related to socioeconomic conditions, since rural children may be more involved in domestic activities to assist with household chores such as gardening, animal grazing, and fetching water [61]. In another way, children living in urban areas presented higher engagement in sports activities [70], which is also related to motor, physical and social development [101]. These disparities are explained by the higher possibilities to access to structured activities in urban areas, as well as the diversity of practices, sometimes available in extra school time [6,7,8]. Further, considering the physical structure of urban areas, the lack of available, free of taxes, and secure spaces for children usage increase the search for structured spaces [7]. These differences are also expressed when analysing the weekly physical activity time, in which children living in rural areas tend to present higher levels of physical activity during the evenings [67,80]. Factors such as the perception of security, availability of outdoor spaces for physical activity practice, and even parental availability to play with their children in the outdoor area may be related to these differences [67,80]. Inconsistent findings were shown for active transportation [64,67,88].

Obesity rates were similar among children living in rural and urban areas [70,71,76]. Since obesity is a complex phenotype, related to individual and environmental characteristics [96], a more holistic approach needs to be considered for a better explanation. Food consumption was previously related to obesity in children [102]; however, non-differences were provided regarding the place of residence, and the pattern of food consumption in these different places. Among inactive children, those living in rural areas presented higher levels of being overweight [81]. 

### 4.3. Differences in Sedentary Behaviour within and between Rural and Urban Areas

Among those living in rural areas, girls [37,45,49,54] and older children [53,54,59] spent more time in sedentary behaviour than their peers. Differences in movement behaviours according to sex were already expected. Historical, cultural, economic, and family factors affect the involvement and engagement in physical activities [88], being able to reinforce sexist patterns that more hectic tasks are for boys and calmer for girls. Given that, girls are more prone to perform activities in sedentary positions (such as sitting). Comparing urban and rural children, regardless of sex, urban children spent more time in sedentary behaviour [4,62,65,66,68,78,84,85,90] and were less prone to meet screen time recommendations [62,87]. Economic aspects, such as more access to TV, video games and smartphones, as well as less availability of outdoor spaces to play, can play a relevant role in these differences [65,66]. The WHO Physical Activity and Sedentary Behaviour Guidelines [13] emphasize the association of sitting time/screen with several negative health outcomes, so although it does not stipulate a daily screen time, it is recommended that *“kids and teens should limit the amount of time they spend sedentary, particularly the amount of recreational screen time”*. A revised study on this subject reported that children who spent more time in sedentary behaviours were more likely to be overweight [63].

Two studies, sampling the same subjects but using different instruments for data collection, found distinct results regarding differences in sedentary behaviour between rural and urban girls—data derived from accelerometer showed that urban girls spent more time in sedentary behaviour [74], while data derived from self-reported questionnaire revealed that rural girls were more sedentary (screen time) [75]. This demonstrates the relevance of choosing the measurement method according to the study purpose, avoiding possible bias in the results. Although the use of device-based measurements (such as accelerometer or pedometer) presents a trend of greater reliability for the collected variable [103], they are more expensive and often less accessible in countries with less investment and support for researches, highlighting that the use of questionnaires seems to be a more viable alternative [103]. In addition, Tremblay et al. [104] noted, in their systematic review, that informed information is usually the most used to measure sedentary behaviour, “allowing” to classify subjects based on time spent in screen (the 2 h/day is the most used cut-off point), which can lead to a false conclusion that there is a screen time limit during the day to be, or not be, sedentary.

Regarding screen time, the findings indicate that rural children had more TV time [88]. This fact may be related to a possible difficulty of access to other types of screens (e.g., computer, smartphone, video game), perhaps due to economic factors or, often, difficulties with internet access, which ends up limiting the functionalities of these devices, made less attractive, especially for children [9,88]. Such aspects can also explain the results that showed that children with lower socioeconomic status spent less time in sedentary behaviour [48]. This lack of access to technologies can lead to greater involvement in active play and reduce the possibilities of sedentary leisure [88].

### 4.4. Limitations, Strengths, and Remarks

Limitations of the present study include the search strategy used which, although it was thought to cover most of the studies on the subject studied, may have excluded some pertinent articles from the results, especially those published in languages other than English, Portuguese, and Spanish. In addition, the different forms of measurement and cut-off points of physical activity and sedentary behaviour used, hinder the synthesis and generalization of the findings presented by the studies. In addition, the limited number of reviewed papers that used other health outcomes than BMI does not allow us to deeply understand the relationship between movement behaviour and health outcomes in rural children. Despite these limitations, to the author’s knowledge, this is the first review that has set out to investigate differences in movement behaviours and health outcomes in rural children. In addition, the fact that we used three different languages (Portuguese, English, and Spanish) allowed us to access a larger number of articles for the analysis, which increased the accuracy of our screening process. Therefore, performing a meta-analysis with the information discussed here could increase the robustness of the synthesis of the results presented in the study.

Based on these findings, suggestions for future studies include: increasing the evidence about movement behaviours among children living in South America and Oceania countries, since differences regarding geographic, social, cultural, economic, and political factors are evident; increasing the level of evidence about the role of the school for physical activity in children from rural areas, given the inconsistent findings; provide information about natural (e.g., weather) and built environments (e.g., school structure, bike lanes) for physical activity and sedentary behaviour, especially considering the socioeconomic gradient.

## 5. Conclusions

Our findings showed that rural children were more physically active and spent less time in sedentary behaviour compared to their urban peers. Among the rural samples, boys were more active and had less screen time than girls and, overall, the rates of overweight were between 16% and 38% and did not differ according to the place of residence (rural and urban). As expected, we noted that low levels of physical activity and long periods of sedentary behaviour were associated with negative health outcomes, such as obesity. We observed a greater number of publications in the recent five years, and a greater concentration of research conducted by high-income countries, especially North Americans and Europeans, which were also the ones that used more device-based measurements, although most studies have been conducted using questionnaires. Finally, the methodological quality attributed to most of the studies was considered high.

## Figures and Tables

**Table 1:** Checklist used to evaluate studies’ quality.

Item	Question	Score
1	Was the objective(s) of the study clearly defined(s)?	0–2
2	Were the characteristics of the participants presented in detail in the methods (number of subjects, sex, age, country/city)?	0–2
3	Was the sample size justified?	0–2
4	Were the instruments used clearly described in the methods section?	0–2
5	Were the statistical analyses clearly presented?	0–2
6	Were the results detailed (means and standard deviations and/or change/difference, effect size)?	0–2
7	Were the conclusions appropriate, giving the methods of study and the objectives?	0–2
8	Are there implications for practice given the results of the study?	0–2
9	Were the limitations of the study recognized and described by the authors?	0–2
10	Is there any future direction described by the authors?	0–2
Total		0–20

Note: Adapted from Abarghoueinejad et al. (2021) [23]. Scores: Not = 0; Maybe = 1; Yes = 2.

**Table 2:** Data extracted from articles that sampled only rural children (54.4%).

Continent	Study and Country (Region)	Sample Size	Instrument Used	Main Results	QS (%)
		Age/School Grade			
Africa	Benefice et al., 2001 [25] Senegal (Niakhar)	406 13–14 years	Accelerometer	PA varied throughout the years, in time and intensity; the time spent in sedentary activities and light activities did not change over the years; there was an increase in VPA (from 1998 to 1999).	80
	Nhantumbo et al., 2008 [26] Mozambique (Calanga)	845 7–16 years	Questionnaire	Girls presented higher means in recreational games; boys had higher means than girls in participation in sports activities; girls were more active overall and had higher values for domestic activities.	80
	Prista et al., 2009 [27] Mozambique (Calanga)	256 6–16 years	Accelerometer	Boys had more minutes of VPA than girls; the intensity of PA decreased significantly with age; domestic tasks were the predominant mode of PA.	90
	Croteau et al., 2011 [28] Kenya (West region)	72 8–12 years	Pedometer	The mean daily step was 14,558 ± 3993; boys were more active than girls; 79% of students reported walking or running to school (these children had a significantly higher average of daily steps compared to those who used other ways of transport).	75
	Craig et al., 2013 [29] South Africa	89 7, 11, 15 years	Accelerometer	The average time spent in MVPA was low: MVPA/day was higher for boys (7, 11, 15 years old) than for girls. Overall, participants had relatively high levels of PA.	90
	Santos et al., 2013 [30] Mozambique (Calanga)	209 7–15 years	Questionnaire	Boys spent less time in PA than girls; high levels of PA and low prevalence of METs were observed.	85
	Minnaar et al., 2016 [6] South Africa	78 5–14 years	Pedometer	20% of the children were overweight or obese (62.5%, girls); Boys were more active in transportation than girls; children between 9 and 11 years took an average of 11,601 steps per day; non-significant associations were found between physical activity and BMI.	90
Asia	Cheah et al., 2014 [31] Malaysia (Sarawak)	145 13–15 years	Pedometer	11.7% of children were obese and 15.2% were overweight; boys recorded a higher average of steps per day compared to girls; the level of total PA was low.	90
	Williams et al., 2016 [32] Nepal (Eastern region)	399 6–18 years	Accelerometer	Girls spent more time in sedentary behaviour than boys; at the ages of 13 and 14 years boys were more sedentary than girls during the week; boys were involved more in MVPA; children were more active during non-school days and teens were more active during school days.	75
	Abe et al., 2020 [33] Japan (Unnan)	1794 9–15 years	Questionnaire	20.1% of the children reached the recommended levels of MVPA; boys were more likely to achieve the MVPA guidelines; children who liked to practice physical activity were more likely to meet the MVPA recommendations.	85
	Bin Saad et al., 2020 [34] Malaysia (Kuching/Samarahan)	227 4–6 years	Questionnaire	About 46% of children were physically active; among obese children, 70% were inactive; inactive children had more screen and video game time; older children were twice more likely to be physically active than their 4 year old peers.	90
	Zhang et al., 2020 [35] China (Mojiang)	2264 12–15 years	Questionnaire	The prevalence of overweight/obesity was 8.0% in the sample; adolescents who did not comply with the recommendations for PA and screen time presented a higher risk of overweight/obesity than those who fulfilled both recommendations.	85
	Fukushima et al., 2021 [36] Japan (Unnan)	821 3–6 years	Questionnaire	66.2% of children achieved the PA guidelines at early childhood; boys were more active than girls; only 75 children and 42 children reported spending 60 min/day practicing PA during week days and weekend days, respectively.	80
Europe	Kelly et al., 2005 [37] Ireland (Carlow)	41 4–5 years	Accelerometer	Irish children spent 19% of their time in LPA; boys spent more time in LPA; girls spent 2% of their time in MVPA, while boys spent 4%; girls spent 82% of the monitored time in sedentary behaviour while boys spent 74%.	85
	Ciesla et al.,2014 [38] Poland	25,816 6–7 years	Questionnaire	20.8% of boys and 18.2% of girls were overweight; boys played more hour at computer; among physically inactive children most were girls, while boys were the majority among those who practiced within the MVPA recommendations	90
	Gutierrez et al., 2015 [39] Spain (Cuenca)	956 10–12 years	Questionnaire	Girls were more sedentary and had a higher percentage of body fat; 27.6% of children were overweight and 7.2% had high levels of cardiometabolic risk factors; 69% attended school walking or cycling; boys, children with normal weight and without cardiometabolic risk lived closer to school.	85
	Machado-Rodrigues et al., 2016 [40] Portugal	254 13–16 years	Accelerometer	21% of boys and 24% of girls were overweight; boys spend more time in PA and MVPA and girls spend more time in sedentary behaviour during the week.	100
	Jonczyk et al., 2021 [41] Poland (Silesia)	589 10–13 years	Questionnaire	26.3% of the children were obese and 16.4% were overweight; more than 70% of the children went to school on foot; 36% dedicated less than 3 h/week to physical activity; a significant correlation between BMI and the number of hours/weeks spent on physical activity were observed among 10 year old girls and 11–12 year old boys;	65
North America	Pate et al., 1997 [42] USA (South Carolina)	361 10–11 years	Questionnaire	32.1% of the children were inactive; students who watched TV or played video games for ≥3 h/day were more likely to be inactive; Girls were twice more likely to be classified as less activity than boys.	90
	Harrell et al., 2003 [43] USA (North Carolina)	1211 11–14 years	Questionnaire	Children whose parents had low socioeconomic status reported less sedentary activities than their peers; boys had higher levels of VPA than girls.	85
	Davy et al., 2004 [44] USA (Scott County)	205 11 years	Pedometer	22% were at risk of overweight and 32% were overweight; BMI and average daily steps did not differ between boys and girls.	80
	Treuth et al., 2005 [45] USA (Maryland)	229 7–19 years	Accelerometer	Older girls had more fat mass than younger girls; boys had higher accelerometer counts compared to girls; higher body fat was associated with higher time spent on sedentary activities in girls; and lower body fat was associated with more time spent on LPA.	80
	Moore et al., 2008 [46] USA (Georgia)	116 4th, to 11th grade	Questionnaire	Children with high levels of PA reported more video game time on school days compared to their peers. Children with low levels of PA were three times more likely to be positive for metabolic syndrome and 2.4 times more likely to be overweight compared to those with high levels of PA.	85
	Glover et al., 2011 [47] USA (South Carolina)	98 6–11 years	Questionnaire	Approximately 55% of children practiced some sport; 66% watched TV for more than 2 h on school nights; 50% of participants used computers for about 2 h during the week.	65
	Newton et al., 2011 [48] USA (Louisiana)	272 4th to 6th grade	Accelerometer	Boys had 8 min more MVPA/day than girls; African-American children with low socioeconomic status had 36 min less sedentary behaviour compared to African-American children with average socioeconomic level.	90
	Shriver et al., 2011 [49] USA (Oklahoma)	237 8–10 years	Questionnaire	38% of children were overweight or obese; almost 30% of children spent 60 min in MVPA the day before; children who watched TV the day before (79.3%) reported approximately 1 h watching; boys had more TV and computer time than girls; obese children spent less time in MPA and MVPA than their non-obese peers.	90
	Limbers et al., 2014 [50] USA (Texas)	189 8–19 years	Questionnaire	23.9% of the children had excess body fat; girls were more likely to have excess body fat; PA and sedentary behaviour were not significant predictors for excess adiposity.	90
	Cottrell et al., 2015 [51] USA (Virginia)	566 5–15 years	Questionnaire	31% of the children were overweight (14.8%) or obese (16.2%); children’s PA was significantly higher among low-income families than in all other categories.	85
	Chow et al., 2016 [52] Canada (Saskatchewan)	69 3–5 years	Accelerometer	The group that received PA and feeding intervention was involved significantly more in MVPA; sedentary behaviour decreased after the intervention.	85
	Daly et al., 2017 [53] USA	153 9–11 years	Accelerometer	50% of the children were overweight or obese; 6th graders spent more time in sedentary behaviour (79.2%) than 3rd, 4th and 5th graders; 3rd (7.8%) and 5th graders (7.0%) participated more in MVPA than 6th graders (5.0%); children involved in MVPA had lower BMI, and children who spent more time in sedentary behaviour had higher BMI	75
	Button et al., 2020 [54] Canada (Ontario)	134 8–14 years	Accelerometer/ questionnaire	On average, children spent 7.4 h/day in sedentary behaviour; boys spent, on average, less 28.91 min daily in sedentary behaviour than girls; increasing age, the time spent in sedentary behaviour increases (14.37 min each year).	85
	Brazendale et al., 2021 [55] USA (Florida)	54 6–11 years	Accelerometer/ questionnaire	22.2% of the children were overweight/obese; children had 75 ± 44 min of MVPA and 615 ± 154 of sedentary behaviour; children were less sedentary on school days than on the weekend; children accumulated less screen time on school days vs. weekdays.	90
	Button et al., 2021 [56] Canada (Ontario)	90 8–14 years	Accelerometer/ questionnaire	Boys had 26.49 min more MVPA than girls; for each increase of 1 ºC Canadian children reached 1.18 min more MVPA; children reached 24.38 min less MVPA on rainy days	95
	Kellstedt et al., 2021 [57] USA (Nebraska)	418 3rd to 6th grade	Questionnaire	6th-grade children had lower mean MVPA compared to their 3rd- and 5th-grade peers; boys reported more minutes of MVPA than girls; children who participated in sports had more average daily minutes of MVPA than those who did not participate.	90
Oceania	Barnett et al., 2002 [58] Australia (North)	231 3rd and 4th grade	Questionnaire	Similar levels of school physical activity between sexes; 33.2% of girls and 34.8% of boys were involved in MVPA; 9.2% of girls and 10% of boys were involved only in VPA; overall MVPA was 36.2%, and exclusively vigorous activity was 12.9%.	65
South America	Fronza et al., 2015 [59] Brazil (Santa Catarina)	294 10–19 years	Questionnaire	The prevalence of obesity was 20.4%; associations were observed only between TV time and age during weekdays; associations between excessive TV viewing during weekend days and the variables studied were not observed.	90
	Valdés-Badilla et al., 2015 [60] Chile (Temuco)	23 7–12 years	Questionnaire	13% of children were overweight and 21.7% were obese; children spent 2225.9 min/week in PA, spent 9592.1 METs/week, and remained 228.6 min/week seated; girls had more total physical activity/week than boys.	65

Note: QS (quality score); PA (physical activity); LPA (low physical activity); VPA (vigorous physical activity); MVPA (moderate to vigorous physical activity); MET (metabolic equivalent of task); BMI (body mass index); USA (United States of America).

**Table 3:** Data extracted from articles comparing rural × urban/suburban children (45.6%).

Continent	Study and Country (Region)	Sample Size	Instrument Used	Main Results	QS (%)
		Age/School Grade			
Africa	Christoph et al., 2017 [61] Uganda (Mukono)	148 11–16 years	Questionnaire	Active commuting was common in both locations; activities such as digging, herding animals, and looking for water were high among rural children; in general, the BMI was positively related to the female sex, living in rural area, and report being active a greater number of days/week; girls were less active.	85
	Kidokoro et al., 2021 [62] Kenia (Maasi and Nairobi)	261 10–12 years	Accelerometer	Rural children had lower BMI than urban children; urban children had more TV and computer time; rural children met screen time guidelines to a greater extent, spent less time in sedentary behaviour, had more time in MVPA, and had more step count compared to urban ones.	100
Asia	Xu et al., 2008 [63] China (Nanjing)	6848 12–18 years	Questionnaire	Rural children were less likely to be overweight than urban children; BMI and TV time were positively related only among rural male adolescents; students who watched TV for more than 7 h/week were 1,5 times more likely to be overweight.	85
	Itoi et al., 2012 [64] Japan (Tohoku)	277 11–12 years	Accelerometer	Children in rural areas walked less to school and had lower step counts than urban children; children who had lower step counts/day and shorter walking time to school had higher BMI.	95
	Karkera et al., 2014 [65] India (Mangalore)	650 9–13 years	Questionnaire	Rural children performed more activities before and after school and spent more time in physical activities and less time watching TV compared to urban children	75
	Baygi et al., 2015 [66] Iran	5682 10–18 years	Questionnaire	In the least developed region (Southeast), children aged 10 to 13 years had less screen time. (TV and computer); in addition, regarding MVPA, 55.8% of children from the mentioned region, practiced <1 h/week, 34.0% spent between 1–2 h/week (the highest percentage among regions), and 10.2% spent >2 h/week.	95
	Kundapur et al., 2017 [67] India (Mangalore)	300 12–16 years	Questionnaire	There were differences in physical activity levels between boys; the total score of physical activity among rural adolescents was higher and going to school by bike was more frequent among boys in rural schools compared to their urban peers.	75
	Lu et al., 2019 [68] China (Xangai)	2175 7–12 years	Questionnaire	In the rural group, obesity among boys was 5.4%, while among girls it was 1.7%; the prevalence of children who used computer/watched TV for more than 3 h per day was 8.6% in the rural group, and 27.7% in the urban group; the prevalence of children who spent more than 3 h per day doing homework was 5.3% in the rural group and 21,2% in the urban group.	50
Europe	Loucaides et al., 2004 [69] Chipre	256 11–12 years	Pedometer	During winter, urban children reached more average steps/day than rural children, and the opposite was observed during summer; urban children spent more time playing video games compared to rural children during winter; rural children spent more time outdoors than urban children in both winter and summer; parents of urban children reported transferring their children more often to places where they can be physically active (in winter and summer).	80
	Bathrellou et al., 2007 [70] Chipre	1140 10–12 years	Questionnaire	Rural children spent more time in PA (of any kind) after school and reported being busier with outdoor tasks during the week; rural children played fewer sports than urban children; time spent in MVPA and screen time did not differ between urban and rural children; the prevalence of normal weight, overweight and obesity did not differ between rural and urban children.	80
	Ara et al., 2007 [71] Spain (Aragon)	1068 7–12 years	Questionnaire	The prevalence of overweight and obesity was similar between rural and urban children; physical activity did not differ according to the place of residence (rural or urban).	70
	Bounova et al., 2010 [72] Greece (Evrytania)	542 11–13 years	Questionnaire	Suburban girls achieved significantly lower VPA Scores than urban boys and rural boys; 57% of all subjects practiced MVPA for three days for more than 60 min; boys from suburban districts scored higher in VPA; for MVPA, rural children scored more than suburban per day; both rural and suburban adolescents tend to be less active on Sunday.	85
	Craggs et al., 2011 [73] United Kingdom (England)	1653 9–10 years	Accelerometer	The percentage of parents with higher education was greater among children from rural areas; mean level of physical activity differed between sex; no differences were found between environments; regardless of location, boys were more active than girls; overweight children were less active compared to normal-weight children; in addition to sex and overweight indexes, the preference for PA also showed associations with total PA.	100
	Machado-Rodrigues et al., 2012 [74] Portugal	362 13–16 years	Accelerometer	18.0% of the children were overweight and 3.9% were obese; the prevalence of overweight and obesity was 17% and 5.1% among rural youth, and 20% and 1% among urban youth, respectively; rural boys spent more time in LPA during the week, while urban boys spent more time in MVPA at the weekend; urban girls spent more time in sedentary activities, less time in LPA and MVPA compared to rural girls; MVPA was positively correlated with cardiorespiratory fitness among rural and urban adolescents; adolescents with higher levels of cardiorespiratory fitness had a lower relative risk of being overweight and/or obese than young people classified as normal-weight.	100
	Machado-Rodrigues et al., 2014 [75] Portugal	362 13–16 years	Accelerometer	Urban boys spent less time in physical inactivity than rural boys, but no differences between groups on weekend days were observed; urban boys had less time in sedentary activities; rural boys spent more time in LPA during the week, while they spent more time in MVPA over the weekend; urban girls spent less time in sedentary activities and less time in LPA; urban girls spent less time in MVPA every day.	90
	Morais et al., 2017 [76] Portugal (Vila de Rei)	129 10–12 years	Questionnaire	There was little difference between adolescents who spent more than 2 h/day watching TV/playing video games (66.1%, urban; 80.0%, rural); more rural students did not perform other activities besides physical education classes at school; rural children walked more; living in a rural environment was not an independent predictor of overweight or obesity, or to increase the percentage of body fat.	80
	Franco et al., 2020 [77] Spain (Badajoz)	542 11–13 years	Questionnaire	During primary education students were moderately active, both rural and urban; in secondary education, urban children were more moderately active; children from rural areas practiced more MVPA; girls were more inactive in both environments; in secondary education, boys from the rural area were the most inactive; students in urban areas were more moderately and very active.	75
	McCrorie et al., 2020 [78] United Kingdom (Scotland)	774 10–11 years	Accelerometer	Rural children spent more minutes/day in LPA; urban children spent more minutes/day in sedentary activity; urban children had higher levels of MVPA during spring and significantly lower levels in winter, compared to their rural peers.	95
North America	Felton et al., 2002 [79] USA (South Carolina)	1668 12–14 years	Questionnaire	Among black girls, those from rural areas practiced more VPA than urban girls; among white girls, those from urban areas were more vigorously active than those from rural areas; physical activity, in general, did not differ between urban and rural areas.	70
	Joens-Matre et al., 2008 [80] USA (Lewa)	3416 8–12 years	Questionnaire	The prevalence of overweight was higher among rural children; rural children were 1.47 times more likely to be overweight than children from small towns; urban children were less active; boys were more active than girls; urban children also reported less activity after school and at night than children in rural areas.	80
	Liu et al., 2008 [81] USA	44,631 10–17 years	Questionnaire	Rural children were more likely to be overweight; among physically active children, rural children were more likely to be overweight; urban children were more likely to be physically inactive than rural children; urban girls were more likely to be physically inactive than rural girls; rural children were 21% less likely to be physically inactive than urban children.	90
	Liu et al., 2012 [82] USA	14,332 2–19 years	Questionnaire	The prevalence of overweight/obesity was higher among rural children; percentages of excessive total screen time did not differ between urban and rural children; rural children aged 2 to 11 years had higher odds of being overweight; among adolescents aged 12 to 19 years, the rural population was more likely to be overweight/obese.	90
	Moore et al., 2014 [83] USA (North Carolina)	804 4th–8th grade	Accelerometer	In boys, there were non-differences in MVPA/day between the urbanity categories; rural girls had more MVPA/day time and were more likely to accumulate more than 60 min MVPA/day compared to suburban and urban girls.	85
	Cristiana et al., 2021 [4] USA	1128 12–17 years	Questionnaire	27.1% of rural adolescents were overweight; MVPA did not differ between rural and urban adolescents; rural adolescents presented a low screen time and a low probability of engaging in active commuting.	100
Oceania	Aucote et al., 2009 [84] Australia (Ballarat)	393 10–12 years	Questionnaire	All subjects were more likely to engage in more than the recommended 14 h of small-screen sedentary activity per week; metropolitan children spent more time playing video games than regional and rural children; socioeconomic status was not a significant predictor of BMI in the studied sample.	85
	Hodgkin et al., 2010 [85] New Zealand	2375 5–15 years	Questionnaire	Rural boys and girls had lower values for subcutaneous fat compared to their urban peers; children living in urban areas had lower levels of physical activity compared to those with better socioeconomic status; among Maori children, urbans were more active than rural ones; screen time was lower in rural children, but significantly different only at ages 5–7.	90
	Dollman et al., 2012 [86] Australia	2071 9–16 years	Questionnaire/ pedometer	Urban adolescents were less active than rural adolescents; urban youth reported lower MVPA; urban girls had lower MVPA than those from remote regions; daily steps were lower among boys and girls from major cities than those from peripheral regions; urban boys had more screen time per day than those from remote areas; TV time was shorter among boys from remote areas; among girls, total screen and TV time did not differ between categories.	90
	Bell et al., 2016 [87] Australia (South Region)	4637 9–11 years	Questionnaire	36.7% of rural children practiced 60 min or more of PA/day and 18.8% spent more than 2 h on electronic devices, and both values were higher compared to their peers from urban areas; rural children were more likely to perform physical activity and comply with screen time recommendations than urban children; patterns of healthy lifestyle behaviours were significantly higher among rural children.	90
South America	Andrade Neto et al., 2014 [88] Brazil (Vitória and Santa Maria de Jetibá)	1242 7–10 years	Questionnaire	Active commuting, regardless of the destination, was more frequent among urban children; rural children watched more TV but used less video games and computers, as well as had more average daily commuting time on foot or by bike to school; being female, living in urban areas, and being overweight was the profile of children who did not reach the recommendation of 300 min of physical activity/week.	90
	Lizana et al., 2016 [89] Chile (Valparaíso)	363 8–13 years	Questionnaire	The prevalence of obesity was higher among urban girls; more than 90% of the children did not reach 7 h of MVPA/week; boys perform more PA than girls, regardless of the place of residence; the prevalence of obesity was 30.88% in urban children and 28.93% in rural children.	90
	Flor-Garrido et al., 2016 [90] Equator (Paute)	314 12–19 years	Questionnaire	The prevalence of overweight was lower in rural children; rural children spent fewer hours doing homework, fewer hours of physical/unplanned activity, and fewer screen hours.	95

Note: QS (quality score); PA (physical activity); LPA (light physical activity); MVPA (moderate to vigorous physical activity); BMI (body mass index); USA (United States of America).

## Data Availability

Not applicable.
